# Supplementary material for: Formulation Study of a Co-Processed, Rice Starch-Based, All-in-One Excipient for Direct Compression Using the SeDeM-ODT Expert System
Source: Pharmaceuticals (Basel). 2021 Oct 14;14(10):1047. doi: 10.3390/ph14101047 (PMC8537138; doi:10.3390/ph14101047)
Supplement: Supplementary file 1 [file pharmaceuticals-14-01047-s001.zip › pharmaceuticals-1415678-supplementary.pdf]

Supplementary material

Article

Formulation Study of a Co-processed, Rice Starch-Based, All-in-One Excipient for Direct Compression Using SeDeM-ODT Expert System

Karnkamol Trisopon[1], Nisit Kittipongpatana [1,2], Phanphen Wattanaarsakit[3], and Ornanong Suwannapakul Kittipongpatana [1,2*]

1 Department of Pharmaceutical Sciences, Faculty of Pharmacy, Chiang Mai University, Chiang Mai 50200, Thailand

2 Research Center for Development of Local Lanna Rice and Rice Products, Chiang Mai University, Chiang Mai 50200, Thailand

3 Department of Pharmaceutics and Industrial Pharmacy, Faculty of Pharmaceutical Science, Chulalongkorn University, Bangkok 10330, Thailand

* Correspondence: ornanong.sk@gmail.com; Tel.: +66 53 944397

Pearson correlation coefficients for the relation of the SeDeM-ODT parameters

SeDeM-ODT parameters	Da	Dc	Ie	Ic (%)	ICD	IH	α	t''	HR (%)	H (%)	Pf (%)	Iθ	DE	DCD	DSD
Da															
Dc	0.98**														
Ie	-0.86*	-0.76*													
Ic (%)	-0.04	0.14	0.53												
ICD	-0.40	-0.52	-0.06	-0.68											
IH	0.06	-0.12	-0.55	-1.00**	0.67										
α	0.21	0.08	-0.62	-0.77*	0.71	0.77*									
t''	0.26	0.16	-0.59	-0.61	0.68	0.60	0.95**								
HR (%)	0.03	0.08	0.22	0.33	-0.77*	-0.33	-0.53	-0.66							
H (%)	-0.12	-0.13	0.18	-0.01	-0.50	0.00	-0.35	-0.52	0.90**						
Pf (%)	0.34	0.26	-0.43	-0.44	-0.10	0.44	0.24	0.25	0.17	0.41					
Iθ	-0.21	-0.09	0.39	0.60	0.06	-0.60	-0.16	-0.03	-0.32	-0.63	-0.87*				
DE	0.43	0.33	-0.71	-0.44	-0.09	0.46	0.61	0.06	0.31	0.34	-0.07	-0.30			
DCD	0.46	0.38	-0.69	-0.33	-0.13	0.34	0.55	-0.01	0.30	0.25	-0.19	-0.16	0.99**		
DSD	0.50	0.41	-0.75	-0.40	-0.16	0.42	0.56	-0.07	0.36	0.33	-0.08	-0.27	0.99**	0.99**	

** Correlation is significant at the 0.01

* Correlation is significant at the 0.05

Da = Bulk density

Dc = Tapped density

Ie = Inter-particle porosity

IC = Carr index

Icd = Cohesive index

IH = Hausner ratio

α = Angle of repose
t'' = Powder flow
%HR = Loss on drying
%H = Hygroscopicity
%Pf = Particle < 50
Iθ = Homogeneity index
DE = Effervescence
DCD = Disintegration time with disk
DSD = Disintegration time without disk
